# De Novo Genome Assembly of Chinese Plateau Honeybee Unravels Intraspecies Genetic Diversity in the Eastern Honeybee, *Apis cerana*

**DOI:** 10.3390/insects12100891

**Published:** 2021-10-01

**Authors:** Lan Lan, Peng Shi, Huali Song, Xiangyou Tang, Jianyang Zhou, Jiandong Yang, Mingxian Yang, Jinshang Xu

**Affiliations:** 1 College of Life Sciences, Chongqing Normal University, Chongqing 401331, China; cqnull@aliyun.com (L.L.); cqnushipeng@163.com (P.S.); shlcqnu@163.com (H.S.); 2018110513041@stu.cqnu.edu.cn (X.T.); jyzhou24@163.com (J.Z.); 2 Engineering Research Center of Biotechnology for Active Substances, Ministry of Education, Chongqing 401331, China; 3 College of Animal Sciences and Technology, Sichuan Agricultural University, Chengdu 611130, China; yangjd@sicau.edu.cn (J.Y.); mx.yang@sicau.edu.cn (M.Y.)

**Keywords:** *Apis cerana*, genome, gene loss/gain, chemosensory receptors, immunity

## Abstract

**Simple Summary:**

In this study, we obtained a chromosome-scale assembly genome of *Apis cerana abansis,* which lives in the southeastern margin of the Titan Plateau, by using PacBio, Illumina and high-throughput chromatin conformation capture (Hi-C) sequencing technologies. With a more comprehensive annotation pipeline, we obtained an ampler and more accurate *Apis cerana* genome than previous studies. Comparative genomic analysis was performed to identify the divergence among different *A. cerana* genomes by studying two aspects: the differential content of repeat content and the gene loss/gain events occurred in chemosensory receptors and immune-related proteins. Our results show that the content of repetitive sequences differ in types and quantity among four *A. cerana* strains; the gene loss/gain events in chemoreceptor- and immune-related proteins occur in different *A. cerana* strains, especially in *A. cerana abansis* (Aba strain). Specifically, while compared with the other three published genomes, the Aba strain contains the largest number of repeat contents and loses the largest number of both chemosensory-receptor- and immune-related proteins, as well as subfamilies, whereas the Baisha strain contains the largest number of chemoreceptor- and immune-related proteins. We hypothesized that gene loss/gain may be evolutionary strategies used by the different *A. cerana* strains to adapt to their respective environments.

**Abstract:**

*Apis cerana abansis*, widely distributed in the southeastern margin of the Qinghai-Tibet Plateau, is considered an excellent model to study the phenotype and genetic variation for highland adaptation of Asian honeybee. Herein, we assembled and annotated the chromosome-scale assembly genome of *A. cerana abansis* with the help of PacBio, Illumina and Hi-C sequencing technologies in order to identify the genome differences between the *A. cerana abansis* and the published genomes of different *A. cerana* strains. The sequencing methods, assembly and annotation strategies of *A. cerana abansis* were more comprehensive than previously published *A. cerana* genomes. Then, the intraspecific genetic diversity of *A. cerana* was revealed at the genomic level. We re-identified the repeat content in the genome of *A. cerana abansis*, as well as the other three *A. cerana* strains. The chemosensory and immune-related proteins in different *A. cerana* strains were carefully re-identified, so that 132 odorant receptor subfamilies, 12 gustatory receptor subfamilies and 22 immune-related pathways were found. We also discovered that, compared with other published genomes, the *A. cerana* *abansis* lost the largest number of chemoreceptors compared to other strains, and hypothesized that gene loss/gain might help different *A. cerana* strains to adapt to their respective environments. Our work contains more complete and precise assembly and annotation results for the *A. cerana* genome, thus providing a resource for subsequent in-depth related studies.

## 1. Introduction

Asian honeybee *Apis cerana* is one of the ten *Apis* species wildly distributed throughout the Asian region [1]. The geographic isolation and biological diversity lead to significant genetic differences and differential adaptation of *A. cerana* to the varied habitats [2,3,4]. The native honeybee from the Aba prefecture of the Chinese Western Sichuan Plateau, named *Apis cerana abansis* [5,6,7], is distributed on the southeast edge of the famous Qinghai-Tibet Plateau. *Apis cerana abansis* (also called the Aba strain in this study) presents distinguishable morphological characteristics compared to other ecological types of Asian honeybees. The morphological indexes of the Aba strain, such as proboscis length, the area of the forewing and the third and the fourth notum are larger than other *A. cerana* strains [7]. Moreover, recent studies have proved that honeybees in the Western Sichuan Plateau form a distinct genetic group compared with other geographic strains, due to the highland geographic isolation [2,3,7,8]. Hence, *Apis cerana abansis* is considered to be an excellent model to study the phenotype and genetic variation for the highland adaptation of Asian honeybee.

A complete and accurate genome assembly is a crucial basis for studying the connection between gene function and the characteristics of organisms and is necessary for functional genomics and population genomics studies [9]. Second-generation sequencing technology is the most cost-effective due to high-throughput, low-cost features, allowing for the bulk sequencing of samples; however, its sequencing read length is limited due to technical reasons [10,11]. Third-generation sequencing technology does not require PCR amplification and can effectively avoid systematic errors caused by biases in PCR amplification. Moreover, the fragment length of DNA sequences in a single run is very long, up to 10,000 bp, which is very helpful for assembling repetitive sequences and genome assembly [12,13]. The Hi-C method uses chromosome conformation capture to identify chromosome interactions that can be used to group and construct alleles using their physical proximity in the genome [14,15]. Each of these technologies has its shortcomings, and none of them can produce an optimal assembly on its own.

So far, three *A. cerana* strains, GenBank assembly accession as GCA_001442555.1, GCA_002290385.1 and GCA_011100585.1 (labeled as Korea, Wufu and Baisha in this study), which were respectively collected from Korea; Wufu Mountain of Jiangxi, China; and Baisha of Hainan Island, China, have been sequenced [16,17,18]. The second-generation sequencing technique was applied to both the Korea strain and the Wufu strain genomes, whereas the Baisha strain was sequenced using third-generation sequencing technology. Compared to *Apis mellifera* genomes, *A. cerana* has fewer odorant receptors, as well as a more effective social defense system, such as physiological resistance to the parasitic mite Varroa [16,17]. A chromosome-scale assembly of the *A. cerana*, Baisha strain genome is 215.67 Mb in size (examined using PacBio and Hi-C sequencing technology) with a contig N50 of 4.49 Mb, which provides high-quality data for genomic comparison with other *Apis* species [18]. In *A. mellifera*, a high-quality genome was assembled by first generated contigs based on PacBio sequencing, which were then merged with linked-read 10× Chromium data, followed by scaffolding using a BioNano optical genomic map and a Hi-C chromatin interaction map, complemented by a genetic linkage map [19].

Repeat content can occupy a large proportion of the eukaryotic genome [20], and its amplification provides a broad source of variation for genome evolution [21]. Currently in *A. cerana*, repeat content has not been systematically identified. The current large variation in repeat content contained in different genomes (from 4.2% to 9.65%) [16,17,18] is not conducive to a more in-depth understanding of the repeat content contained in *A. cerana*.

The odorant receptor is an important basis and prerequisite for the olfactory recognition system of insects, and it is an essential element for the mediation of the conversion of chemical signals such as external odorants into electrical signals [22], while gustatory receptors play an important role in daily behaviors such as feeding and reproduction in insects [23]. From previous studies, we know that Wufu contains 81 olfactory receptors and 10 gustatory receptors [16], Korea contains 119 olfactory receptors and 13 gustatory receptors [17], and Baisha has not been systematically identified [18]. In *A. mellifera*, a total of 170 olfactory receptors and 10 taste receptors were identified [24].

The honeybee has been widely used as a model animal for studying individual and population immunity [25]. In early research, almost 160 immune-related proteins were identified in Korea and 144 immune-related proteins were identified in Wufu, so that scholars believe that the immune system of *A. cerana* contains 12 mainly immune-related pathways [16,17]. The immune-related proteins of Baisha have not been systematically predicted. Like all insects, honeybees lack an adaptive immune system, so it is important to study the bee’s immune system [18].

Several genomes of *A. cerana* have been sequenced, thus giving us a better understanding of the genomic organization of this species. However, genomic diversity and variation within different ecological types of this species remain poorly understood. According to previous studies, Korea, Wufu and Baisha strains collected from different geographic locations can be approximately considered representative for three ecological types of Changbaishan, Central China and Hainan Island. Therefore, complete genomic sequences of those three strains give a good opportunity to delineate the intra-species ecological genomics of the Asian honeybee, *A. cerana*.

In this study, we used third-generation sequencing technology, second-generation sequencing and Hi-C sequencing technology to sequence the collected *A. cerana* Aba strain from the Western Plateau of Sichuan and obtain a high-quality highland *A. cerana* reference genome. We demonstrated the divergence of different *A. cerana* genomes by studying two aspects: the differential content of repeat content, the gene loss/gain events that occurred in chemosensory receptors and immune-related proteins. Gene loss/gain can contribute to evolutionary novelties and can be positively selected [26,27]. Thus, we hypothesized that in *A. cerana*, gene loss/gain may contribute to environmental adaptation and could result from a positive selection.

## 2. Materials and Methods

### 2.1. Genome Sequencing and Assembly

The *A. cerana* workers from the plateau region in Western Sichuan were collected from Mosudu Village, Songgang Town, Markang City, Aba Prefecture, Sichuan Province (102°10′38″ E; 31°50′45″ N; approximately 2600 m above sea level).

The genomic DNAs were extracted from the bee’s thorax through conventional the CTAB (cetyltrimethylammonium bromide) [2,28] method. Total RNA was extracted from total bee tissues but without intestine to avoid microorganism pollution.

Three different genomic DNA libraries were constructed and sequenced according to the manufacturer’s instructions to generate a high-quality chromosome-scale assembly genome: (i) 20-kb library using a PacBio Sequel platform, (ii) Hi-C reads sequencing by phase genomics, and (iii) paired-end library using Illumina NovaSeq PE150 platform (150 bp sequencing length with insert size of 350 bp). Two different cDNA libraries were constructed and sequenced according to the manufacturer’s instructions to generate a complete and accurate cDNA data that greatly contributed to genomic annotation: (i) 4-kb library using a PacBio Sequel platform, (ii) paired-end library using Illumina NovaSeq PE150 platform (150 bp sequencing length with insert size of 400 bp). All sequencing was conducted by the Novogene Bioinformatics Institute, Beijing, China.

The PacBio reads were assembled into high-quality consensus sequences by Wtdbg2 v2.5 [29] with the ‘-k 0 -p 21 -K 1000.049988 -A -S 4.000000 -s 0.070000 -g 0 -X 50.000000 -e 3 -L 0’ setting. Racon v1.3.1 [30] (-u -t 40) and Pilon v1.22 [31] (-Xmx300G --diploid --threads 8) were used as a polishing tool after the assembly by the Illumina paired-end reads. The paired-end reads from Hi-C were used to cluster, order and orient the draft contigs onto chromosomes by BWA v0.7.8 [32] and were further assembled into chromosome level using Hi-C proximity ligation data by ALLHIC 0.9.8 [33].

The quality of the assembled genome was assessed by BUSCO v3.0.2 [34] and CEGMA v2.5 [35] with default parameters. The Aba was set as a reference, and the draft genomes of Korea and Wufu were elevated to chromosome-scale assemblies using ragtag v2.0.1 [36]. The structure collinearity analysis was performed by using Mummer4 [37] with default parameters.

### 2.2. Genome Annotation

A combined strategy based on homology alignment and de novo search to identify the whole genome repeats was applied in our repeat annotation pipeline. Ab initio prediction was used to build de novo repetitive elements database by RepeatModeler v1.73 [38] with default parameters for building the raw transposable element (TE) library. A custom library (a combination of Repbase and our de novo TE library) was supplied to RepeatMasker v4.0.9 [38] with -s parameters for DNA-level repeat identification. The homolog prediction was performed using RepeatMasker and the Repbase (20181026).

Homologous proteins for homolog prediction were downloaded from Ensembl and NCBI. The protein sequences of *Apis mellifera* (ncbi.GCF_003254395.2), *Apis cerana* (ncbi.GCF_001442555.1), *Nasonia vitripennis* (ensembl.metazoa.v32), *Polistes dominula* (ncbi.GCF_001465965.1), *Cephus cinctus* (ncbi.GCF_000341935.1), *Athalia rosae* (ncbi.GCF_000344095.1), *Apis dorsata* (ncbi.GCF_000469605.1) and *Apis florea* (ncbi.GCF_000184785.3) were aligned to the assembly genome using TblastN v2.9.0+ [39] (E-value ≤ 1e−5). Then, we predicted the exact gene structure of the corresponding genomic regions on each TblastN hit by GeneWise v2.4.1 [40].

For ab initio gene prediction, Augustus v3.2.3 [41], Geneid v1.4 [42], GlimmerHMM v3.04 [43] and SNAP (2013-11-29) [44] were used in our gene prediction pipeline with self-trained model parameters.

The RNAseq reads from Illumina sequencing were assembled by Trinity v2.8.5 [45] for gene structure annotation; the reads from PacBio sequencing were filtered by the SMRT IsoSeq analysis process for sequencing data quality testing. The filtered RNAseq reads were compared to the genome by GMAP (2017-11-15) [46], and the gene structure was annotated by PASA v2.4.1 [47].

The non-redundant reference gene set was generated by merging genes predicted by three methods with EvidenceModeler v1.1.1 [48], using PASA terminal exon support and including masked transposable elements as input into gene prediction.

Gene functions were assigned according to the best match by aligning the protein sequences to the Swiss-Prot using Blastp (with a threshold of E-value ≤ 1e−5). The motifs and domains were annotated using InterProScan v5.31 [49] by searching against publicly available databases, including PRINTS [50], Pfam [51], PANTHER [52] and PROSITE [53]. The Gene Ontology (GO) IDs for each gene were assigned according to the corresponding InterPro entry.

### 2.3. Identification of Chemoreceptor Proteins and Immune-Related Proteins

All odorant receptors of *Drosophila melanogaster* were collected from FlyBase [54]. The odorant receptor proteins of homo species, *Bombyx mori*, and mice were downloaded from NCBI [55]. The odorant receptor proteins of *A. florea* and *A. mellifera* were obtained, referring to another past study [24,56], as well the predicted odorant receptor proteins of two *A. cerana* strains. Ultimately, a total of 6535 predicted odorant receptor proteins from our collected protein sets were used as a database for the prediction of odorant receptor proteins of different *A. cerana* strains in this study. Almost 101 gustatory receptor proteins were obtained and set as a database to identify the gustatory receptor in different *A. cerana* strains. These gustatory receptors were from the FlyBase [54], while NCBI collection included the gustatory receptor proteins of *Drosophila melanogaster*, *Bombyx mori*, *A. mellifera* and different *A. cerana* strains that were predicted and reported in previous studies [57]. A total of 999 proteins involved in immune-related pathways from *A. mellifera*, *Drosophila melanogaster* and *Bombyx mori* were collected through the KEGG [58] database and were set as a database to identify the immune-related proteins contained in different *A. cerana* strains. The different *A. cerana* strains were predicted by the protein2genome (--percent 40, *n* = 1) module of Exonerate v 2.4.0. We discarded the proteins with translation errors due to the presence of stop codons. The longest protein located in the same gene loci was taken as the prediction result; the putative gene-containing regions recognized from the BLAST hits but not through good Exonerate hits were separately re-examined.

The predicted odorant receptor needed to contain the 7tm_6 domain to be considered an odorant receptor protein [56]. The gustatory receptor with four transmembrane regions “6tm”, “ICL3”, “7tm” or “Trehalose_recp”, was considered a gustatory receptor protein [59,60]. Those odorant receptor proteins and gustatory receptor proteins were aligned by MAFFT v7.455 [61] separately, and the tree was built by IQ-TREE2 [62] with “-m MFP -B 1000 -bnni”. Bootstrap analysis was performed using 1000 replicates.

We use Orthofinder v2.3.8 [63] to get 1:1 orthologous gene. Genes showing evidence of positive selection along each branch were identified with the branch-site model by PAML v4.7 [64], by setting different *A. cerana* strains as the foreground branch. Likelihood ratio test (LRT) indicated that the model allowing sites to experience specific adaptations provided a significantly better fit to the data than the null model, in which sites could under positive selection. The positively selected genes must contain the positive selection sites.

## 3. Results

### 3.1. Genome Assembly and Gene Annotation

Genome sequencing of the Aba strain using PacBio provided a total data size of 128.98G (515.92X), reads number of 8,451,522, an average length of 15,262 bp, N50 with 21,185 bp. Genome sequencing using Illumina sequencing provided a total size of 36.54G (151.76X). Transcriptome data through PacBio sequencing were: total data size of 31.51G, reads number of 28,246,937, the average length of 1116 bp, N50 with 1350 bp; Illumina sequencing: total data size of 25G (Table S1). In order to assemble contigs into scaffolds further, the total size of 36,536,558,700 bp raw data of Hi-C reads was generated.

The total size of the assembled contigs was 226.97 Mbp, contig N50 of 7.91 Mbp; the total size of the scaffold was 226.99 Mbp, and the scaffold N50 was 13.28 Mbp. We also obtained 16 pseudochromosomes that represent the 16 chromosomes of *A. cerana abansis* (Figure S1) with a length range from 7.07 Mbp to 26.41 Mbp and 867 unplaced scaffolds with a total length of 11,377,790 bp (5.01% of the genome).

Among the 1658 BUSCO groups searched, 1636 BUSCO groups were identified (98.7 percent of the total BUSCO groups) (Table S2). The conserved genes in six eukaryotic model organisms (248 genes in total) were selected to constitute the core gene library. According to the results of CEGMA evaluation, 248 CEGs (core eukaryotic genes) were assembled out of 244 genes, accounting for 98.39%. Both the results of BUSCO and CEGMA supported the high quality of the genome assembly.

De novo prediction, homology-based and transcriptome-based strategies were combined to identify and annotate protein-coding genes (Table S3). Finally, 11,240 genes with an average CDS length of 1533 bp and an average transcript length of 8256 bp were predicted in Aba. The number of exons per gene is 6.18, and mean exon length is 248 bp. The mean length of introns is 1298 bp, much longer than that of exons. Using different databases to annotate those predicted proteins, we found that 93.7% of the genes could be annotated (Table S4).

### 3.2. Genome Comparison between Different A. cerana Genome

The Aba genome has a contig N50 of 7.91 Mb and a scaffold N50 of 13.27 Mb, which are larger than contig N50 (3.89 Mb) and the scaffold N50 (13.17 Mb) of Baisha (Table 1). Meanwhile, the contig N50 was higher than in *A. mellifera*. However, the number of scaffolds not mounted was higher in Aba (867) than in Baisha (110). Baisha has the smallest genome size (215.67 Mb) in *A. cerana,* and Wufu (228.79 Mb) has the largest genome size in *A. cerana*. Aba predicted the greatest number of genes at 11,240.

Based on the predicted longest transcript proteins of different *A. cerana* strains, 832 new proteins were identified in Aba compared with the other three *A. cerana* strains, including 785 proteins of unknown function. Baisha showed 775 newly identified proteins compared with the other three *A. cerana* strains, of which 748 were of unknown function. Korea showed 215 new proteins not predicted in other *A. cerana* strains, of which 192 proteins were of unknown function. Wufu had 130 new proteins not predicted in the protein set of other *A. cerana* strains, of which 119 proteins were of unknown function (Table S5).

We identified one common chromosomal structural variant among Aba and Baisha, as well as *A. mellifera,* by collinearity analysis (Figures S2 and S3). A previous study identified 4 chromosomal structural variants in Baisha compared with the *A. mellifera* [18]. However only 1 chromosome structure variation was identified between Aba and *A. mellifera* (Scaffold id: “NC_037649.1”), which confirms that at least one structural variant is presented between these two species, *A. cerana* and *A. mellifera*.

### 3.3. Repeat Content in Different A. cerana Strains

Through our repeat annotation pipeline, 31,320,299 bp of Aba genomic sequences were identified as repetitive sequences, accounting for 13.79% of the total genomic length. In a previous study, a total length of 19.73 Mb (9.15%) [18], 14.79 Mb (6.48%) [17] and 9.61 Mb (4.2%) [16] of repetitive sequences were found in Baisha, Korea and Wufu, respectively. A previous study suggested that *A. cerana* had less repeat content than *A. mellifera* [19]. However, our data indicated that Aba contained more repetitive sequences than *A. mellifera* (7.5%, 17.42 Mb). As this was unexpected, we re-identified the repeat content in Baisha, Korea and Wufu.

**Figure 1:**
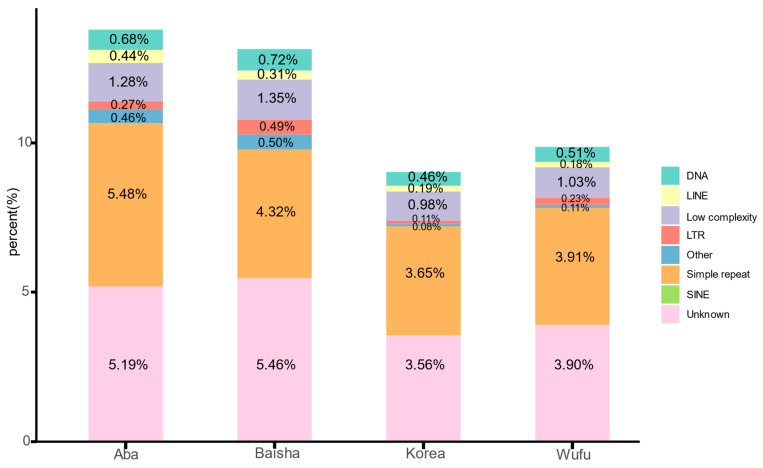
The proportion of different repeat classes in different *A. cerana* genomes.

We found that Aba contained the highest repeat content, followed by Baisha (13.14%, 28.3 Mb), Wufu (9.87%, 22.5 Mb) and Korea (9.02%, 20.6 Mb) (Table S6, Figure 1). All *A. cerana* strains contain more repetitive sequences than *A. mellifera* and have fewer repetitive sequences compared to *B. terrestris* (14.8%, 36.2 Mb) and *B. impatiens* (17.9%, 44.6 Mb) [65].

The repeat classes of Simple repeat and Unknown occupied a larger proportion of repetitive sequences in *A. cerana*, and the repeat classes of DNA, long interspersed nuclear element (LINE), low complexity and short interspersed nuclear element (SINE), were similar in amount in each genome (Figure S4). Among them, we found that in Baisha, the Simple repeat accounted for the lowest proportion of total repetitive sequences, and long terminal repeat (LTR) accounted for a higher proportion of total repetitive sequences than Aba strains.

We found that some repeat classes were more abundant in the genomes based on second-generation sequencing technologies (Korea or Wufu) than in the genomes based on third-generation sequencing technologies (Aba or Baisha), such as L1, L2 in LINE and acro in Satellite.

Interestingly, one PIF-Harbinger of DNA transposon and two centers of Satellite were only presented in Korea. With regards to LTR class, we found that ERVL-MaLR only existed in Baisha and Korea, and there were 1 and 4 copies, respectively. In the SINE class, we found that one tRNA-RTE element was only present in Baisha and Wufu. All the detailed information can be seen in Table S6.

We showed the distribution of the repeat content on the genomes of different strains (Figure S5A,B). We found that the distribution of repeat content on different *A. cerana* strains was consistent, and in the areas with higher repetition content, GC content was lower.

### 3.4. Chemoreceptors in Different A. cerana Strains

**Figure 2:**
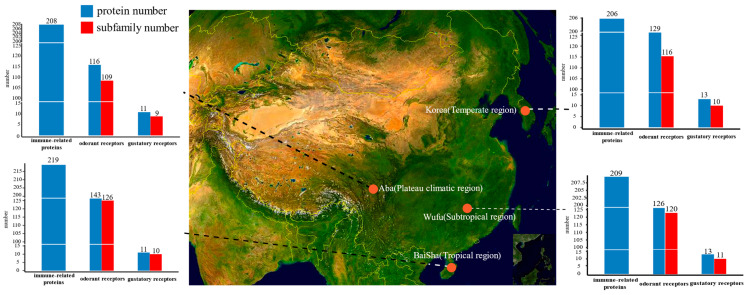
Sample collection sites for each *A. cerana* genome and the type of climate in which they are located. The bar histogram indicates the number of chemoreceptors and subfamilies identified in the different strains and the number of their immune-related proteins.

**Figure 3:**
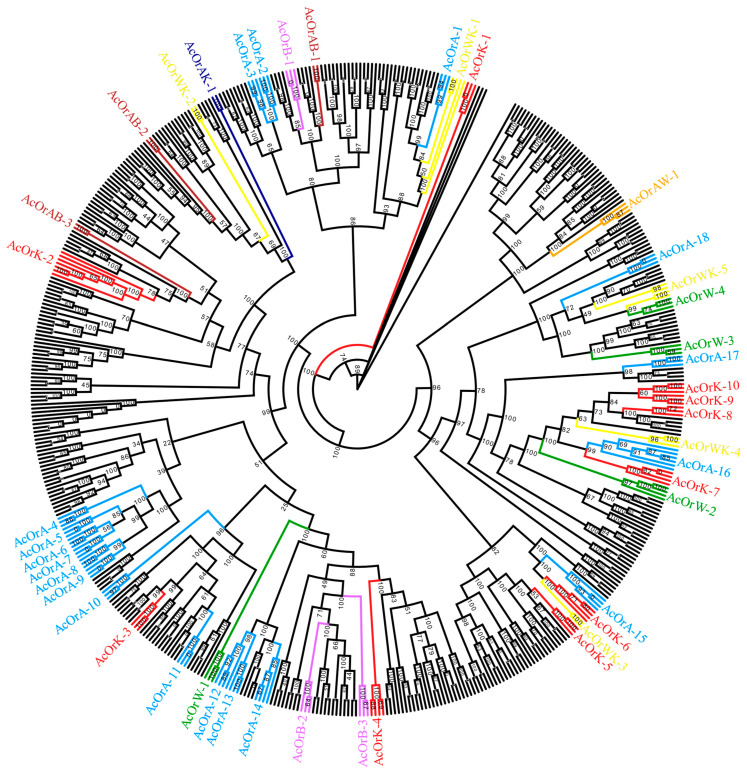
Phylogenetic tree constructed by odorant receptors from different *A. cerana* strains. Blue regions indicate only Aba lost (labeled AcOrA); purple regions indicate only Baisha lost (labeled AcOrB); red regions indicate only Korea lost (labeled AcOrK); green regions indicate only Wufu lost (labeled AcOrW); orange regions represent both Aba and Wufu lost (labeled AcOrAW); yellow regions indicate both Wufu and Korea lost (labeled AcOrWK); dark-blue regions represent both Aba and Korea lost (labeled AcOrAK); brown regions represent both Aba and Baisha lost (labeled AcOrAB).

There were 116 odorant receptors found in Aba, 143 in Baisha predicted, 129 in Korea and 126 in Wufu (Figure 2, Table S7). A total of 132 odorant receptor subfamilies were found in *A. cerana* (Figure 3). Among those, 23 were not detected in Aba, 6 in Baisha, 12 in Wufu and 16 in Korea. In term of these 132 subfamilies, 23 subfamilies were not detected in Aba, 6 in Baisha, 12 in Wufu and 16 in Korea. One subfamily (named AcOrAW-1) was absent simultaneously in Aba and Wufu; three subfamilies (named AcOrAB-1, AcOrAB-2, AcOrAB-3) were absent simultaneously in Aba and Baisha; one subfamily (named AcOrAK-1) was commonly absent in Aba and Korea; five subfamilies (named AcOrWK-1, AcOrWK-2, AcOrWK-3, AcOrWK-4, AcOrWK-5) were not found in Wufu or Korea.

**Figure 4:**
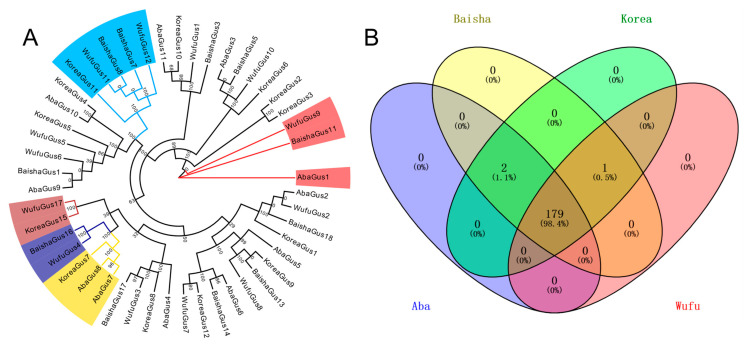
(**A**) Phylogenetic tree constructed by gustatory receptors from different *A. cerana* strains. Blue regions indicate only Aba lost; brown regions represent both Aba and Baisha lost; dark-blue regions indicate both Aba and Korea lost; yellow regions represent both Wufu and Baisha lost; red regions indicate only Korea lost. (**B**) The immune-related KEGG orthologs were identified in different *A. cerana* strains.

Our results showed 11 gustatory receptors in Aba, 11 gustatory receptors in Baisha, 13 gustatory receptors in Wufu and 13 gustatory receptors in Korea (Table S8). After constructing a phylogenetic tree with the gustatory receptors of different *A. cerana* strains (Figure 4A), we found that *A. cerana* contained a total of 12 gustatory receptor subfamilies, of which Aba missed 3 gustatory receptor subfamilies, Korea missed 2 gustatory receptor subfamilies, Baisha missed 2 gustatory receptor subfamilies and Wufu missed 1 gustatory receptor subfamily. Among them, Aba and Baisha missed 1 common gustatory receptor subfamily, Aba and Korea missed 1 common gustatory receptor subfamily and Wufu and Korea missed 1 gustatory receptor subfamily. In addition, Korea has 1 gustatory receptor subfamily specific to different *A. cerana* strains.

### 3.5. Immune-Related Proteins in Different A. cerana Strains

Here, regarding immune-related protein, we re-identified 208 proteins in Aba, 219 proteins in Baisha, 206 proteins in Korea and 209 proteins in Wufu. The immune-related proteins identified in all four strains covered 22 reported immune-related pathways (Table S9). Beside these known pathways, ten immune-related pathways not reported previously were found firstly in *A. cerana* (Table S9), including “Intestinal immune network for IgA production”, “IL-17 signaling pathway”, “Th17 cell differentiation”, “Th1 and Th2 cell differentiation”, “C-type lectin receptor signaling pathway”, “Toll and Imd signaling pathway”, “Neutrophil extracellular trap formation”, “Platelet activation”, “Complement and coagulation cascades” and “Hematopoietic cell lineage”.

By KEGG annotation, we found that Aba missed the immune- related protein annotated as K03009 (DNA-directed RNA polymerases I, II and III subunit RPABC4), while Wufu missed the immune-related proteins annotated as K06061 (mastermind) and K05700 (vinculin) (Figure 4B). K03009 participated in the Cytosolic DNA-sensing pathway, which is involved in functions related to bacterial recognition [66,67]. K06061 participated in the pathway of Th1 and Th2 cell differentiation; Th1 cells primarily mediate cellular immunity, and Th2 cells primarily mediate humoral immunity; this pathway is involved in the signaling process during the differentiation of these two cells [68]. K05700, participated in the “Leukocyte transendothelial migration pathway”, which is involved in the migration of leukocytes to ensure that they can move to specific locations [69].

## 4. Discussion

In this study, PacBio and Illumina sequencing technology were used to assemble and annotate the *A. cerana abansis* genome. Based on a more comprehensive annotation pipeline, more proteins were identified in *A. cerana* compared to previous studies [16,17,18]; we obtained an ampler and more accurate *A. cerana* genome than previous studies, and our results are useful for the in-depth comparative genomic analysis of honeybees.

We identified more repeat contents in *A. cerana* and found no direct correlation between genome size and repeat content in *A. cerana*. Baisha showed the smallest genome size among the different *A. cerana* strains and more repeat content, while Wufu showed the largest genome size but lower repeat content. Additionally, our results showed that the number of repeat contents in *A. cerana* (from 9.02% to 13.79%) was higher compared to *A. mellifera* (7.5%) [19]. It is worth mentioning that the genomes of Aba and Baisha identified more repetitive sequences than Korea and Wufu, indicating that third-generation sequencing technologies can help find more repetitive sequences, which is consistent with the results of a related study of *A. mellifera* [19].

In this study, strains of Baisha and Wufu come from tropical and subtropical regions [16,18], respectively; the Aba strain is mainly distributed in the Qinghai-Tibetan plateau region, and the strain from Korea is distributed in the relatively higher latitude of Korea [18] (Figure 2). The different natural environment influences the abundance and flowering period of the local plants [70,71], which is the vital factor for honeybee living since honeybees closely depend on the local flower nectar and pollen resource. According to the plant phenology observation data set of the China Ecosystem Research Network [8,72], we found that the plant richness and the average annual flowering period in the plateau region were lower than those in non-plateau regions. We speculate that, in the plateau environment (Aba), there are fewer nectar and pollen plants compared to other regions; thus, honeybees in this area have fewer chemoreceptors. On the other hand, Baisha is distributed in tropical areas and has more plant resources than other regions. In order to visit more flowers for nectar collection, honeybees need to recognize the relevant odors. As expected, the highest number of odorant receptors and fewer missing odorant receptor subfamilies were found in Baisha. Interestingly, Korea and Wufu had similar odorant receptors, but Korea was missing more subfamilies. Meanwhile, Aba and Korea shared only one missing odorant receptor subfamily, which suggested that the loss occurred in different subfamilies. Korea is located at higher latitudes compared to Wufu and Baisha, and the vegetation types have greater differences compared to the habitats of Wufu and Baisha. Moreover, the vegetation of Baisha and Wufu at low latitudes is more abundant than that of Korea at high latitudes. We believe that Korea lost some odorant receptor subfamilies during a long adaptation period while some odorant receptor subfamilies increased.

A mutually beneficial synergy has developed between bees, which take in nutrients by feeding on plant pollen and nectar, and plants, which pollinate through bees [16]. As plants have evolved mechanisms to attract and reward bees, bees no longer need to recognize the substances and toxins of numerous plants. Bees are fed by nurse bees from a young age and do not need gustatory sensation to locate and recognize food. In addition, they can recognize objects by their antennae instead of gustatory sensation [24]. We believe that the loss/gain of gustatory receptors in bees could also result from adaptation to their environment. Interestingly, we found the same number of gustatory receptors in Baisha and Aba and in Wufu and Korea. Yet, the loss/gain of gustatory receptor subfamilies was not consistent with the loss/gain of odorant receptor subfamilies. For example, Baisha lost two gustatory receptor subfamilies, but Wufu lost only one gustatory receptor subfamily; Korea had a unique gustatory receptor subfamily.

Temperature and moisture can affect microbial species diversity [73,74]. The area where Baisha honeybees were collected is more suitable for the survival and reproduction of various micro-organisms due to its warmer temperature and humid environment. Other *A. cerana* strains derived from a cold and harsh environment unlike Baisha and probably were subjected to less immune pressure than Baisha, which caused their immune-associated proteins to be lost over time. Among the immune-related protein loss events, we found that Wufu lost two immune-related KEGG orthologs and Aba lost one immune-related KEGG ortholog. We still need more evidence to prove whether the lost events can influence resistance against diseases. In addition, we also found ten new immune-related pathways of *A. cerana* that were not reported previously, which will help us deepen our study of the immune system of *A. cerana*.

In term of chemosensory receptors and immune-related proteins identified in this study, we found that Baisha had the highest number of proteins (373), followed by Wufu (350), Korea (349) and Aba (332). So, we propose that the gene loss/gain that occurred among different *A. cerana* strains may have resulted in an “adaptive gene loss/gain” event. Several studies have mentioned that gene loss/gain may help organisms to adapt to their specific environment [75,76,77,78,79]. In *Drosophila*, the loss of chemoreceptors such as odorant receptors and gustatory receptors is increased when the diet changes, while some of the chemoreceptors that contribute to the current diet undergo gene duplication and are subject to positive selection [80]. Large-scale RNA interference experiments in *C. elegans* [81,82] and *D. melanogaster* [83] revealed that about 65% and 85% of the genes are dispensable in these two species, respectively; when the loss of a gene causes pathway dysfunction, the organism will choose alternative pathways to ensure the stable performance of the pathway function [84]. The most direct manifestation of “gene gain” events is the increase in the number of genes, which can help individuals adapt to the environment in which they survive [85,86].

In summary, whether the gene loss/gain in *A. cerana* has directly affected the corresponding function, such as olfaction, still needs to be proven.

We calculated the selection pressure between different *A. cerana* strains by the site model and the branching site model by PAML. Our results showed that no genes were subjected to under selection, thus suggesting that the phenotypic difference between Aba and other strains does not occur due to selection pressure on the coding region. Accordingly, future studies need to consider the differential expression of genes, the degree of methylation differences, etc. to resolve the reasons for this phenotypic difference.

## 5. Conclusions

Our study provided an ampler and more precise *A. cerana* genome for honeybees. We found differences in genomic structural features among different *A. cerana* strains, such as the number of chemoreceptors, immune-related proteins and repeat content, which is probably related to adaptation to the local environment. This comparative genomic analysis identified new features of the *A. cerana* genome, revealing that the intraspecific genetic diversity of different *A. cerana* strains and gene loss events may promote the adaptation of different *A. cerana* strains to their respective environments. Strains surviving in different environments may provide an excellent biological model for studying the effects of different environments.

## Figures and Tables

**Table 1:** Summary of sequencing data in *Apis* genome.

Genome Assembly	Aba	Baisha	Korea	Wufu	*Apis mellifera*
Genome size (bp)	226,974,933	215,670,033	228,331,812	228,791,026	225,250,884
Number of scaffolds	16 * + 867(unplaced)	16 * + 110(unplaced)	2431	879	16 * + 161(unplaced)
Scaffold N50 (bp)	13,276,899	13,171,513	1,421,626	1,393,515	13,619,445
Scaffold L50 (bp)	7	7	42	46	7
Number of contigs	1043	214	10,707	21,784	228
Contig N50 (bp)	7,911,546	3,898,192	43,751	21,160	5,382,475
Contig L50 (bp)	11	17	1210	2783	13
Gene number	11,240	10,741	10,608	10,182	9880

* Present pseudochromosomes.

## Data Availability

The assembled genome sequence for *Apis cerana* from Aba in this study have been deposited in National Center for Biotechnology Information (NCBI) under the BioProject number PRJNA738447.

## Supplementary Materials

The following are available online at https://www.mdpi.com/article/10.3390/insects12100891/s1, Figure S1: Heat map of Hi-C contact information of the 16 chromosomes; pixel colors represent different normalized counts of Hi-C links between 100 kb non-overlapping windows for all 16 chromosomes (chr) on a logarithmic scale. Figure S2: Collinearity of chromosomes between Aba and Baisha; the x-axis represents the chromosomes from Aba; y-axis represents the corresponding chromosomes from Baisha, from a-p means different homologous chromosome. Figure S3: Collinearity of chromosomes between Aba and *Apis mellifera*; the x-axis represents the chromosomes from Aba; y-axis represents the corresponding chromosomes from *Apis mellifera*, from a-p means different homologous chromosome. Figure S4: The proportion of different repeat classes in different *Apis cerana* genomes’ repeat content. Figure S5: (A) Distribution of repeat content of Aba (blue) and Baisha (gray) over the genome with circular pseudochromosomes. (B) Distribution of repeat content of Korea (purple) and Wufu (green) over the genome with circular pseudochromosomes. b–d show the distribution of the gene density, GC density and repeat density, respectively. The density is calculated in 10k window size. Table S1: Summary of sequencing data for *Apis cerana abansis*. Table S2: Statistics of the BUSCO assessment. Table S3: Results of gene structure prediction. Table S4: Gene annotation statistical results. Table S5: New proteins in different *Apis cerana* strains. Table S6: Repeat content in different *Apis cerana* genome. Table S7: Odorant receptor in different *Apis cerana* strains. Table S8: Gustatory receptor in different *Apis cerana* strains. Table S9: Immune-related protein in different *Apis cerana* strains; the red highlight shows the ten immune-related pathways not reported previously.
