# Supplementary material for: De Novo Genome Assembly of Chinese Plateau Honeybee Unravels Intraspecies Genetic Diversity in the Eastern Honeybee, Apis cerana
Source: Insects. 2021 Oct 1;12(10):891. doi: 10.3390/insects12100891 (PMC8538478; doi:10.3390/insects12100891)
Supplement: Supplementary file 1 [file insects-12-00891-s001.zip › FigureS1.pdf]

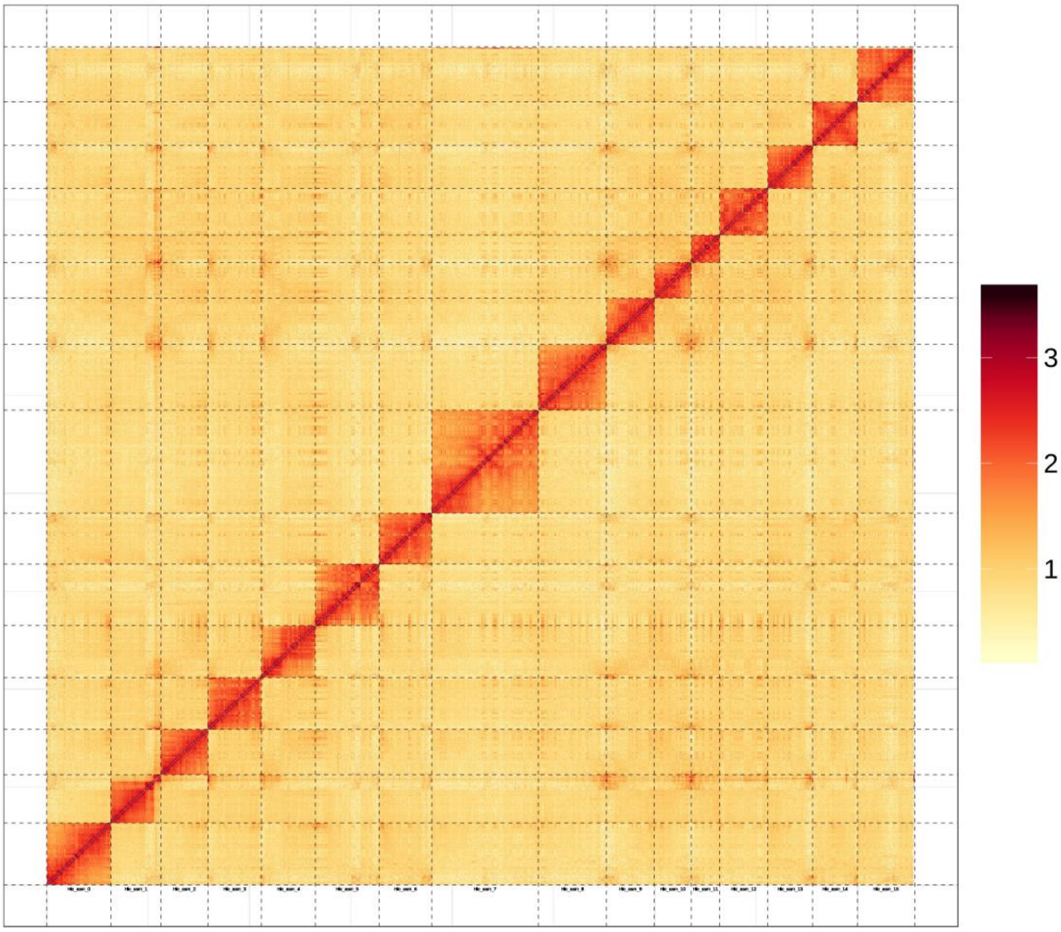

Figure S1. Heat map of Hi-C contact information of the 16 chromosomes, pixel colors represent different normalized counts of Hi-C links between 100kb non-overlapping windows for all 16 chromosomes (chr) on a logarithmic scale.
