# Supplementary material for: De Novo Genome Assembly of Chinese Plateau Honeybee Unravels Intraspecies Genetic Diversity in the Eastern Honeybee, Apis cerana
Source: Insects. 2021 Oct 1;12(10):891. doi: 10.3390/insects12100891 (PMC8538478; doi:10.3390/insects12100891)
Supplement: Supplementary file 1 [file insects-12-00891-s001.zip › FigureS2.pdf]

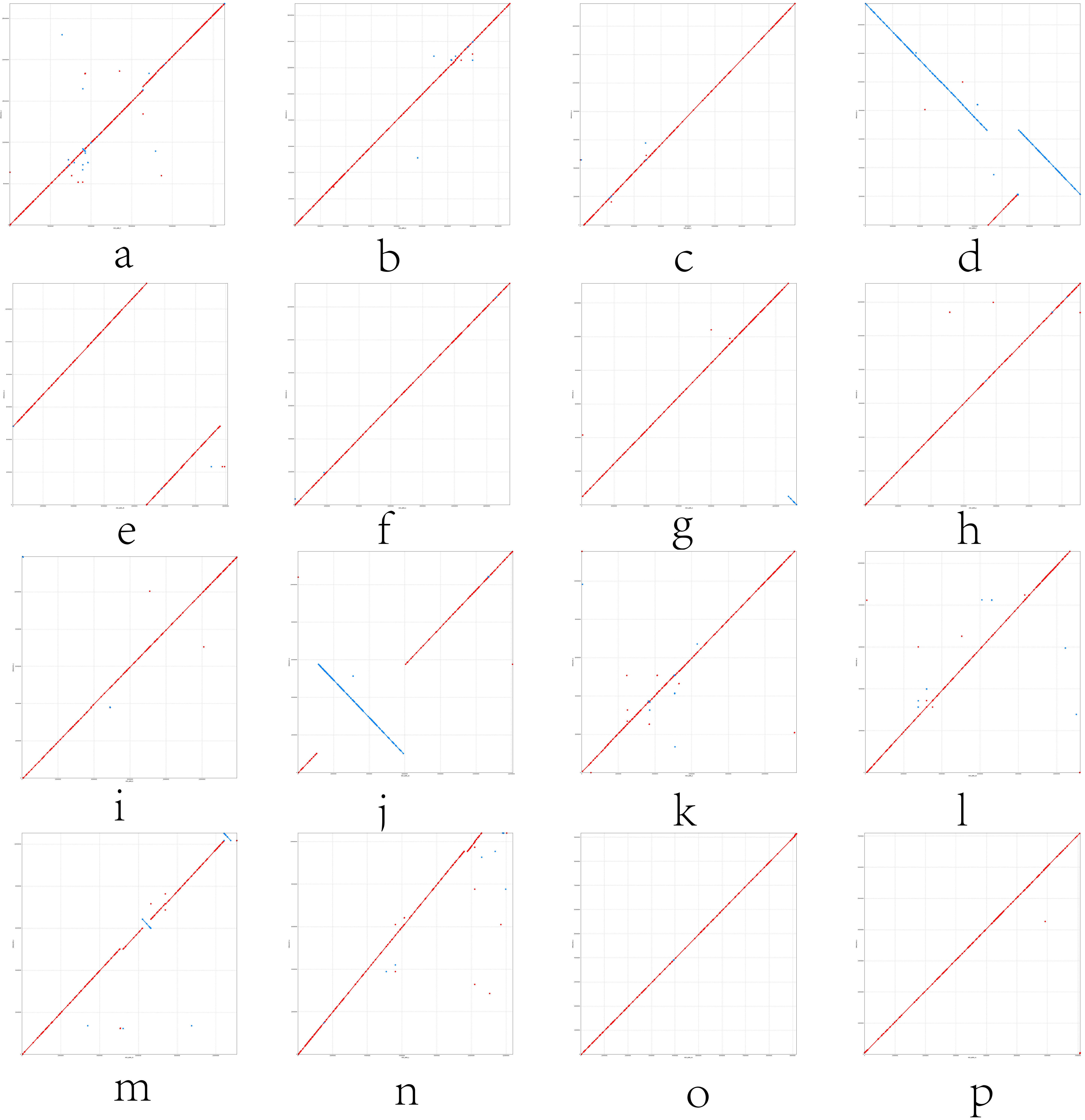

Figure S2. Collinearity of chromosomes between Aba and Baisha, the x-axis represents the chromosomes from Aba, y-axis represents the corresponding chromosomes from Baisha, from a-p means different homol-ogous chromosome.
