# Supplementary material for: De Novo Genome Assembly of Chinese Plateau Honeybee Unravels Intraspecies Genetic Diversity in the Eastern Honeybee, Apis cerana
Source: Insects. 2021 Oct 1;12(10):891. doi: 10.3390/insects12100891 (PMC8538478; doi:10.3390/insects12100891)
Supplement: Supplementary file 1 [file insects-12-00891-s001.zip › FigureS5.pdf]

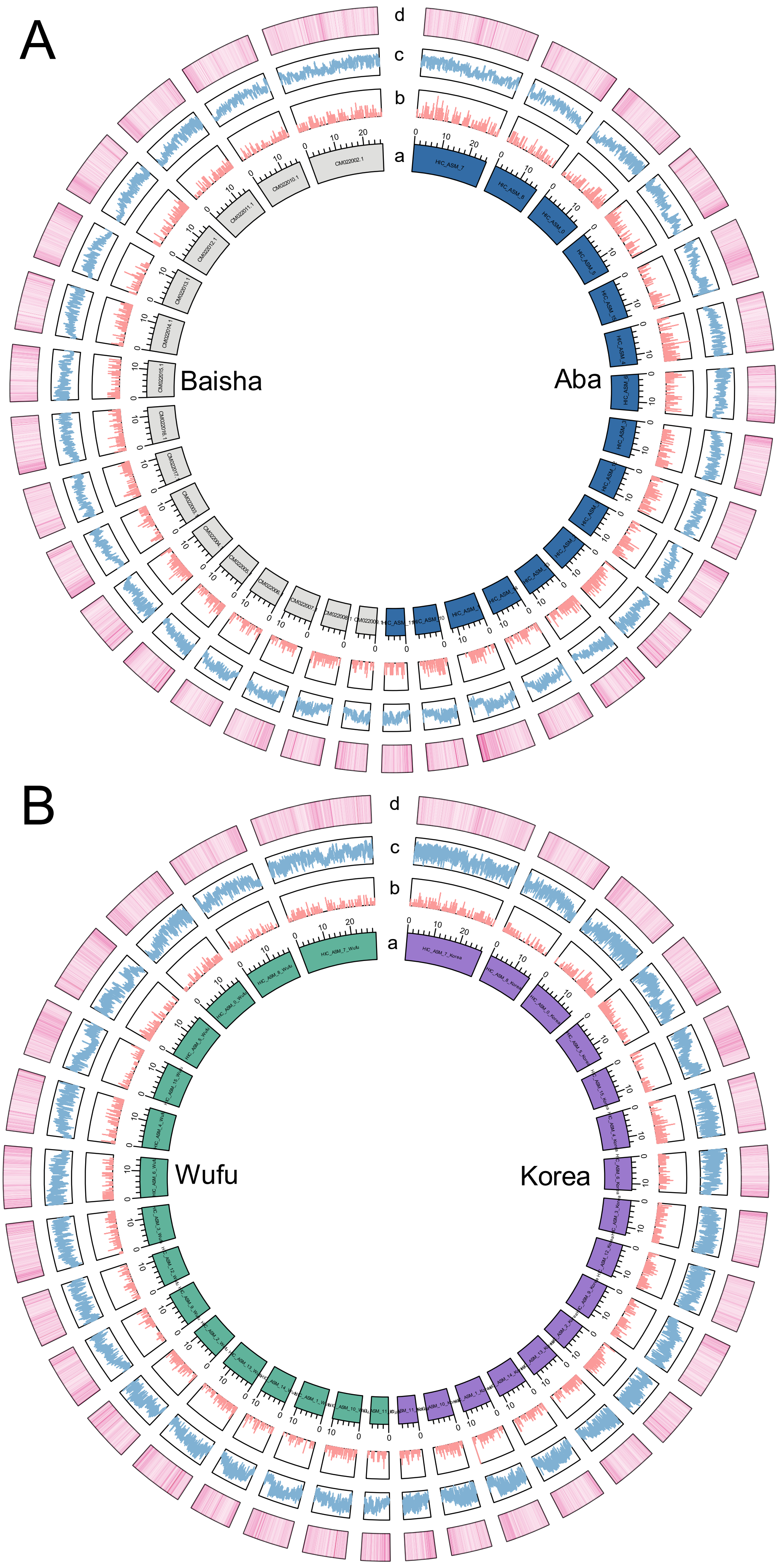

A
d
c
b
a
Baisha
Aba
B
Wufu
Korea

Figure S5. (**A**) Distribution of repeat content of Aba(blue) and Baisha(gray) over the genome with circular pseudochromosomes. (**B**) Distribution of repeat content of Korea(purple) and Wufu(green) over the genome over the genome with circular pseudochromosomes, b–d show the distribution of the gene density, GC density, and repeat density, respectively, with densities calculated in 10k windows.
